# Multiyear analysis uncovers coordinated seasonality in stocks and composition of the planktonic food web in the Baltic Sea proper

**DOI:** 10.1038/s41598-023-38816-0

**Published:** 2023-07-22

**Authors:** Emil Fridolfsson, Carina Bunse, Elin Lindehoff, Hanna Farnelid, Benjamin Pontiller, Kristofer Bergström, Jarone Pinhassi, Catherine Legrand, Samuel Hylander

**Affiliations:** 1 Centre for Ecology and Evolution in Microbial Model Systems - EEMiS, Linnaeus University, 39182 Kalmar, Sweden; 2 Department of Marine Sciences, University of Gothenburg, 40530 Gothenburg, Sweden; 3 GEOMAR Helmholtz Centre for Ocean Research Kiel, E24105 Kiel, Germany; 4 School of Business, Innovation and Sustainability, Halmstad University, 30118 Halmstad, Sweden

**Keywords:** Microbial ecology, Ecology, Environmental sciences, Ocean sciences

## Abstract

The planktonic realm from bacteria to zooplankton provides the baseline for pelagic aquatic food webs. However, multiple trophic levels are seldomly included in time series studies, hampering a holistic understanding of the influence of seasonal dynamics and species interactions on food web structure and biogeochemical cycles. Here, we investigated plankton community composition, focusing on bacterio-, phyto- and large mesozooplankton, and how biotic and abiotic factors correlate at the Linnaeus Microbial Observatory (LMO) station in the Baltic Sea from 2011 to 2018. Plankton communities structures showed pronounced dynamic shifts with recurring patterns. Summarizing the parts of the planktonic microbial food web studied here to total carbon, a picture emerges with phytoplankton consistently contributing > 39% while bacterio- and large mesozooplankton contributed ~ 30% and ~ 7%, respectively, during summer. Cyanophyceae, Actinobacteria, Bacteroidetes, and Proteobacteria were important groups among the prokaryotes. Importantly, Dinophyceae, and not Bacillariophyceae, dominated the autotrophic spring bloom whereas Litostomatea (ciliates) and Appendicularia contributed significantly to the consumer entities together with the more traditionally observed mesozooplankton, Copepoda and Cladocera. Our findings of seasonality in both plankton composition and carbon stocks emphasize the importance of time series analyses of food web structure for characterizing the regulation of biogeochemical cycles and appropriately constraining ecosystem models.

## Introduction

The Baltic Sea is one of the world’s largest brackish systems^1^. It offers multiple socio-economic services, like fisheries, recreational boating, swimming and overall enjoyment of the seascape^2^. The Baltic Sea has been, and still is, heavily influenced by multiple anthropogenic stressors and over exploitation of ecosystem services with severe effects on the ecosystem, e.g., eutrophication and pollution^3,4^. The production of plankton communities is transferred to higher trophic levels. However, the mechanisms regulating the transfer efficiency are still not well defined and additionally, may be affected by anthropogenic stressors^5^. As such, the Baltic Sea can shed light on how ecosystem services, e.g., wild/farmed seafood, removing nutrients and pollutants and tourism, are affected by anthropogenic stressors and thereby offer an expanded view on ecosystem-based management to ensure a sustainable utilization of the Baltic Sea.

Primary producers and consumers like bacterio-, phyto- and zooplankton communities constitute the base of pelagic food webs, together with fungi and archaea, and their metabolic activity has major impacts on global elemental cycling^6–13^. The total flow of energy and matter to higher trophic levels in the food web is highly dependent on microbial community structure and functioning since heterotrophic microorganisms recycle nutrients and dissipate energy produced by primary producers^14–16^. Organisms at the base of the aquatic food web produce biomass and compounds which are important for higher trophic levels throughout the food web. For instance, both hetero- and autotrophic plankton play a key role in the synthesis of e.g. vitamins that are universally needed by higher organisms^17^. Moreover, fish affect the plankton communities, either directly by predation or indirectly by predator-release^18,19^. Therefore, regional long-term investigations of the seasonal dynamics of environmental parameters and bacterio-, phyto- and zooplankton have the potential to provide novel knowledge on the structure and functioning of the food web.

Bacterioplankton play a pivotal role in the aquatic food web, attributable to high abundances, number of species, functional properties, and numerous interactions with other microorganisms as well as higher trophic levels. In aquatic ecosystems, the major primary producers are phytoplankton^20,21^, which only make up 0.2% of the global primary producer biomass, but account for 50% of the net production, e.g.^21,22^. Phytoplankton, in turn are grazed by zooplankton and it is estimated that zooplankton can consume 10–40% of the primary production through grazing daily, depending on the productivity of the ecosystem^23^. Importantly, even small changes in community- or size-structures, and production can have complex and large-scale effects on the entire ecosystem including fish production^4,24,25^. Nevertheless, relatively few studies include multiple trophic levels within the plankton realm, and seldomly cover total biomass estimates along with measures of community composition and abiotic parameters^8,26–32^.

Temporal similarity in community composition can provide insights on the community development, or stability, as shown by Yeh and Fuhrman^32^ in the San Pedro Time Series. Here, the prokaryotic community was found to be quite constant over time whilst the protist community was substantially altered over time^32^. Microbial community composition is affected by interactions with other compartments of the food web (e.g., grazing, viral lysis, allelopathy, symbiosis, competition, and parasitism) as well as abiotic factors (e.g., temperature, water mixing, and nutrient availability). Studies which combine field observations of community composition and biotic and abiotic factors with a correlative approach can serve as a steppingstone in designing mechanistic experimental studies^33^. Here we present long-term and high-frequency data of biotic and abiotic parameters retrieved from the Western Baltic Sea Proper—the Linnaeus Microbial Observatory (LMO)—covering the period 2011–2018. Short-term subsets of some of these data have previously been used to analyze microbial dynamics and seasonal variability as well as micronutrient dynamics, e.g.,^34–41^. In this study, we substantially extend the analysis to bacterio-, phyto- and large mesozooplankton in the planktonic food web over a period of eight years including community dynamics at several trophic levels. The aim of the study is to provide one of the first planktonic food web descriptions of this part of the Baltic Sea and to determine if the seasonal dynamics of the different trophic levels are correlated and to what extent abiotic and/or biotic factors affect the plankton food web.

## Material and methods

### Field sampling

Samples were collected at the Linnaeus Microbial Observatory (LMO; N56°55.8540´, E17°3.6420), located approximately 11 km (6 nautical miles) off the NE coast of the island Öland with a depth of 40 m^34,35,40^. In short, sampling started in March 2011 and was performed with various frequency over the years, from twice weekly to monthly samplings. In the present study, data from samplings performed from 2011-03-25 to 2018-11-27 are included, covering 270 sampling cruises. Water was collected from 2 m depth using a 3 or 5 L Ruttner water sampler at 08.00–10.00 am local time. Temperature and salinity were measured on-site. Water was collected in 10 L, acid-washed, polycarbonate bottles and transported to the laboratory within 1 h. Large mesozooplankton were collected by oblique hauls from the top 30 m using a plankton net (Ø50 cm, 200-μm mesh size) with a fitted flowmeter and stored in a cooling box. Mesozooplankton sampling was included in the sampling regime in November 2013 and sampled monthly. This sampling procedure mainly catch larger sized mesozooplankton and has lower retention of smaller zooplankton such as rotifers, small cladocera, nauplii and other microzooplankton. In addition, starting in 2013, the water column was profiled using a CTD probe (AAQ 1186-H, Alec Electronics, Japan) for temperature, salinity, and light intensity. In the laboratory, samples for nutrients, chlorophyll *a* (Chl *a*), dissolved organic carbon (DOC), colored dissolved organic matter (cDOM), as well as abundance, biomass, and community composition for bacterio-, phyto- and large mesozooplankton were collected and analyzed, see below.

### Abiotic parameters

Inorganic nutrients; nitrate and nitrite (NO_3_^−^ and NO_2_^−^, together presented as nitrate), ammonium (NH_4_^+^), phosphate (PO_4_^3−^), and silicate (SiO_4_^4−^) were collected and frozen until analysis using colorimetric methods (UV-1600 Spectrometer, VWR and DR 5000, Hach Lange) according to Valderama^42^. Detection limits for inorganic nutrients were 0.02 µM for NO_3_^−^ and PO_4_^3−^ and 0.1 µM for NH_4_^+^ and SiO_4_^4−^. Chl *a* was sampled by filtration of ~ 500 mL water in duplicates on A/E filters (Pall Laboratory). Filters were extracted overnight in 96% ethanol in the dark and later analyzed according to Jespersen and Christoffersen^43^, using a Trilogy flourometer (Turner Designs, USA). DOC was sampled in duplicates; 20 mL of water was filtered through precombusted (475 °C, 2 h) GF/C glass fiber filters (nominal pore size ~ 1.2 µm) via gravity, acidified (200 µL 2 M HCl) and stored in precombusted glass vials (475 °C, 3 h) with acid washed lids at 6-8 °C until analysis. DOC content was measured via high temperature catalytic oxidation (HTCO) using a Shimadzu TOC-V analyzer coupled to a TNM-1 unit as well as determined with a Shimadzu TOC 5000 analyzer and a Shimadzu TOC-L Total Organic Carbon Analyzer using an acetanilide dilution series as a standard^44,45^. Detection limit for DOC measurement was ~ 10 µM. cDOM was analyzed by filtering (0.2 µm, Sterivex cartridge filters) a sample into an amber glass bottle, and the absorption coefficients were measured in a 10 cm quartz cuvette over the 420–780 nm range with 2 nm increments in a UV-1600 Spectrometer, VWR^46^. cDOM corresponds to the absorption coefficient at 440 nm.

### Bacterio-, phyto- and mesozooplankton biomass and community composition

Samples for bacterioplankton abundance were preserved with formaldehyde (3.7% final concentration) and kept at − 80 °C until analysis. Samples for total bacterioplankton abundance were enumerated with a flow cytometer (BD FACs Calibur (2011–2012) and Partec Cube8 (2013–2018)) with protocols adapted from Gasol and Morán^47^. Bacterioplankton abundance was converted to carbon biomass using a factor of 20 fg C cell^-1^^48^. Nucleic acids from bacterioplankton for amplicon analysis were collected without prefiltration by filtering up to 10 L seawater on 0.2 µm Sterivex cartridge filters (Millipore). Filters were stored frozen at − 80 °C in TE buffer until DNA was extracted using a phenol–chloroform extraction as described by Boström, et al.^49^ and modified after Bunse, et al.^50^. The V3V4 region of the 16S rRNA gene was amplified using PCRs with the primer pair 341F-805R^51^ as described and validated in Hugerth, et al.^52^. DNA concentrations were analyzed using Qubit 2.0 Fluorometer (Life Technologies) and subsequent gel electrophoresis confirmed amplicon specificity. Sequencing was carried out at the Science for Life Laboratory, Sweden on a MiSeq platform (Illumina, Inc.), producing 2 × 300 bp paired-end reads. For bioinformatical processing, we used the Ampliseq pipeline (https://github.com/nf-core/ampliseq)^53^ using DADA2 to infer amplicon sequence variants (ASVs)^54^. The versions of the software used to produce these results were: nf-core/ampliseq (v1.2.0dev); Nextflow (v20.10.0); FastQC (v0.11.8); MultiQC (v1.9); Cutadapt (v2.8); QIIME2 (v2019.10.0). ASVs were taxonomically annotated against the SILVA database (v132). The resulting amplicon sequence variant (ASV) table was subsequently analyzed in R (see below). Relative abundances were normalized per genome size, using genome “16S rRNA/genome” published by Vetrovsky and Baldrian^55^ and relative abundances represent percentages per taxon. Note that cyanobacteria were deleted from the ASV-generated taxonomy and is only presented as cell counts within the phytoplankton fraction. This is in order to not have this group in both the bacteria and phytoplankton category when analyzing community composition.

Separate samples for phytoplankton and large mesozooplankton abundances were preserved with acidic Lugol’s solution (2% final concentration) and stored in the dark until analyses. Phytoplankton community composition was analyzed in sedimentation chambers according to standard techniques^56,57^ and counted using an inverted microscope (Nikon TMS). Phytoplankton were identified to the genus or species level and cell measurements were used to calculate biovolume and biomass according to Edler^58^ and Olenina, et al.^59^. For the period 2011–2014, size class was not recorded for the phytoplankton counts, therefore the median biovolume and biomass estimates of the size classes was used for the calculations. Starting in 2015, individual size classes were used for the phytoplankton counts. Mesozooplankton were identified to the genus or species level using a stereomicroscope (see table S1) and weights were calculated according to Hernroth^60^, HELCOM^61^ and Sprung^62^. Biomass was estimated from individual wet weight and an assumed carbon content of 5% of the wet weight^63^. As the mesh size of the plankton net was 200 µm, mainly large mesozooplankton are included in the analysis and these were classified into the groups: Copepods, Cladocera, Appendicularia, Rotifers and Other zooplankton (including Thecostraca, Bivalvia, Malacostraca, Gastropoda, Clitellata, Ostracoda, Polychaeta, Thaliacea, Oligotrichea as well as specimens of the phyla Chaetognatha, Echinodermata and unidentified), .

### Data handling, statistical analyses, and graphics

**Figure 1:**
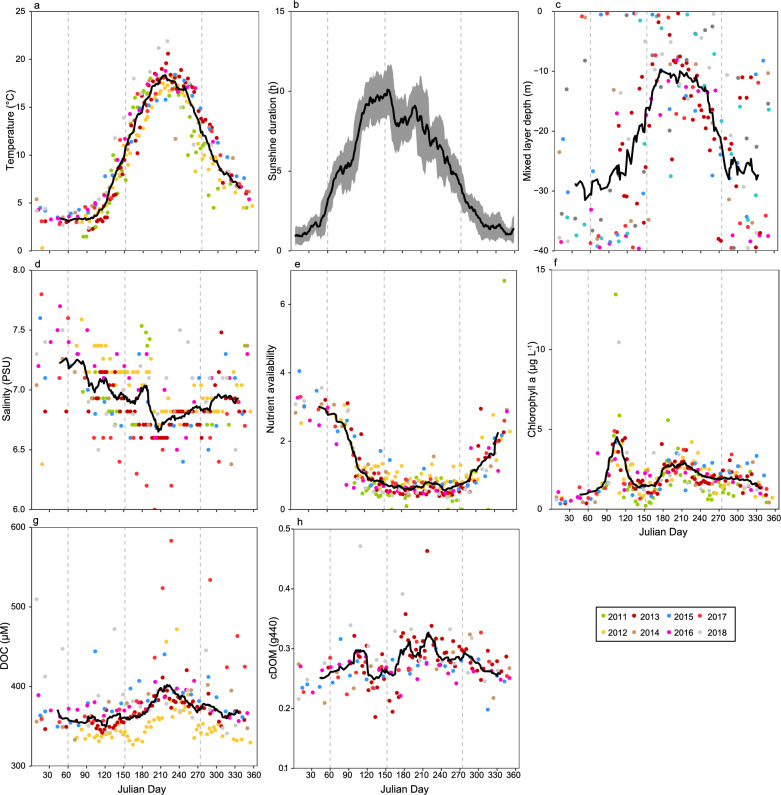
Annual variation of sea-surface temperature (**a**), sunshine duration (**b**) and mixed layer depth (sigma theta 0.125) (**c**), salinity (**d**), nutrient availability (**e**), chlorophyll *a* concentration (**f**), levels of dissolved organic carbon, DOC (g), and colored dissolved organic matter, cDOM (**h**). Grey-shaded area illustrate moving standard deviation and the black line shows the moving average covering 15 julian days, combining all the years. Points illustrate separate samplings over the years with a different color for each year. Vertical dashed lines illustrate breaks for seasons.

All data handling, statistical analyses and graphics were performed using R version 3.6.0^64^ and the “ggplot2” package^11^ in combination with the “gridExtra” package^65^, and the “tidyverse” package^66^. Spearman correlations were performed and visualized using the “psych”^67^ and “corrplot” packages^68^. Spearman's rank correlation coefficient is reported as ρ (rho). If not stated otherwise, values correspond to average values per seasons. Average values correspond to rolling averages covering 15 julian days, spanning all years. If sampling was carried out the same julian day in different years, a mean was calculated prior to calculating the rolling average. The aim of this was to dampen the impact of singular samplings and to acquire an overall pattern. Seasons in the Baltic Proper were defined according to HELCOM as follows; Spring: March–May, Summer: June–September, Autumn: October–December, and Winter: January–February^69^. Bray–Curtis similarity (1- dissimilarity) over time was analyzed using the “vegan” package^70^, using “genus” level in all trophic levels. Non-metric multidimensional scaling (NMDS) analysis was performed using the “*metaMDS()*” function in the “vegan” package^70^ using median community composition to calculate Bray–Curtis dissimilarity to visualize differences in plankton community composition over the study period. Nutrient availability was calculated as described by Fleming and Kaitala^71^, with the equation $\sqrt[3]{\left[{NO}_{3}^{-}+ {NO}_{2}^{-}\right]\times \left[{PO}_{4}^{3-}\right]\times \left[{SiO}_{4}^{4-}\right]}$ where [NO_3_^−^ + NO_2_^−^], [PO_4_^3−^] and [SiO_4_^4−^] are the concentrations of the respective nutrients, to define the combined nutrient level. Mixed layer depth was calculated using the “oce” package^72^, which uses in situ pressure, temperature and salinity to calculate the density (sigma-theta, σθ). Mixed layer depth was defined as the depth where Δσθ > 0.125 kg m^−3^, compared to the surface density^73^. Over the sampling time span, 13 sampling occasions had a mixed layer depth between 0 and 1 m, which could be caused by the stabilization time being too short. However, all samplings are presented as this was considered to have limited effects on the interpretation of our results. In addition, STRÅNG data (sunshine duration and PAR) were extracted from the Swedish Meteorological and Hydrological Institute (SMHI) and produced with support from the Swedish Radiation Protection Authority and the Swedish Environmental Agency. Spearman correlations to investigate relationships among biotic and abiotic parameters used the complete dataset with individual samplings. To investigate relationships among bacterio-, phyto- and the large mesozooplankton community composition, Spearman correlations for relative biomasses of bacterio-, phyto- and large mesozooplankton were used and in addition to the five taxonomical groups of large mesozooplankton presented in Fig. 1. Litostomatea (principally *Mesodinium rubrum*) is a mixotroph^74,75^ but was here considered a predator in the analysis. The output from the analysis was visualized using the “circlize” package^76^ in combination with the “ComplexHeatmap” package^77^. ‘Other zooplankton’ included specimens from the classes Thecostraca, Bivalvia, Malacostraca, Gastropoda, Clitellata, Ostracoda, Polychaeta, Thaliacea, Oligotrichea as well as specimens of the phyla Chaetognatha, Echinodermata and unidentified. Contribution to the relative biomass was always < 2% for each separate class/phyla included in ‘Other zooplankton’.

## Results

### Seasonal variation in abiotic factors

The extensive sampling at LMO revealed strong seasonality for all investigated parameters. In general, surface (2 m) water temperature was stable around 4ºC during winter (January–February, days 0–59), followed by a steady increase from mid-spring (March–May, days 60–151), peaking at ~ 18ºC during summer (June–September, days 152–273), and a decline during autumn (October-December, 274–365) (Fig. 1a). Temperature displayed some interannual variability, e.g. in the range of 4–6 °C among years during summer, but with similar seasonality patterns among the years (Fig. 1a; Fig. S1a). Number of sunshine hours ranged from one to three hours per day in winter to ten hours of sunshine per day in summer during 2011–2019 (Fig. 1b). During winter and autumn, the interannual variation was lower, with ± 1 h, whereas summer values had a larger range among the years with ± 2.5 h (Fig. 1b; Fig. S1b). The water-column was well mixed during winter, until late spring (May) when it started to stratify by temperature which was maintained during summer, followed by well mixed conditions again during autumn (Fig. 1c). Mixed layer depth ranged from 20 to 25 m during winter and autumn but decreased during summer to ~ 10 m. There was a high variability within and among the years for the different seasons, with mixed layer depths ranging from 1 to 38 m during winter, whilst summer values were less variable ranging from 1 to 27 m. In autumn, mixed layer depth varied between 9 and 39 m over the study period (Fig. 1c; Fig. S1c). Salinity was less variable among seasons but was highest during winter and spring and lowest during summer (Fig. 1d) and exhibited some interannual variability (Fig. 1d; Fig. S1d).

The combined nutrient availability ($\sqrt[3]{\left[{NO}_{3}^{-}+ {NO}_{2}^{-}\right]\times \left[{PO}_{4}^{3-}\right]\times \left[{SiO}_{4}^{4-}\right]}$) was high during winter, with a rapid decrease during April (days 91–120) and then remained at a constant level from mid-spring to mid-autumn (October, days 274–304) (Fig. 1e). Nutrient availability showed some interannual variability, with recurring peaks autumn/winter and valleys during summer (Fig. 1e; Fig. S1e). Nitrate displayed the most pronounced seasonality pattern (Fig. S1a) ranging from ~ 2.5 to 0.2 µM. Phosphate showed similar seasonality, ranging from concentrations of 0.8 to 0.1 µM (Fig.S2c). Silicate concentrations ranged from 16 to 8 µM and tended to be higher during winter (Fig. S2d). Ammonium varied between 0.4 and 1.2 with no systematic patterns over time (Fig. S2b). Interannual variability for all nutrients is available in the supplementary files (Fig. S2; Fig. S3). DOC concentrations were at a relatively constant level during autumn, winter, and spring (~ 360 µM) but accumulated after mid-summer (July, days 182–212) reaching up to 410 µM. Towards the end of summer (September, days 244–273), DOC concentrations decreased to levels prior to mid-summer at around 360 µM (Fig. 1g). There was an increase of DOC concentrations in the years 2017 and 2018 which increased the interannual variability (Fig. 1g, Fig. S1g). cDOM was lowest during winter, ~ 0.25 followed by an increase by mid-spring (April, days 91–120) at around 0.30 followed by a decline. A larger and more pronounced peak (~ 0.33) developed around July and lasted until the end of August (days 182–243), by which cDOM decreased to winter values (Fig. 1h). Except for two samplings (spring 2018 and summer 2013), cDOM displayed a low interannual variability (Fig. 1h; Fig. S1h).

### Bacterio-, phyto- and mesozooplankton seasonal dynamics

**Figure 2:**
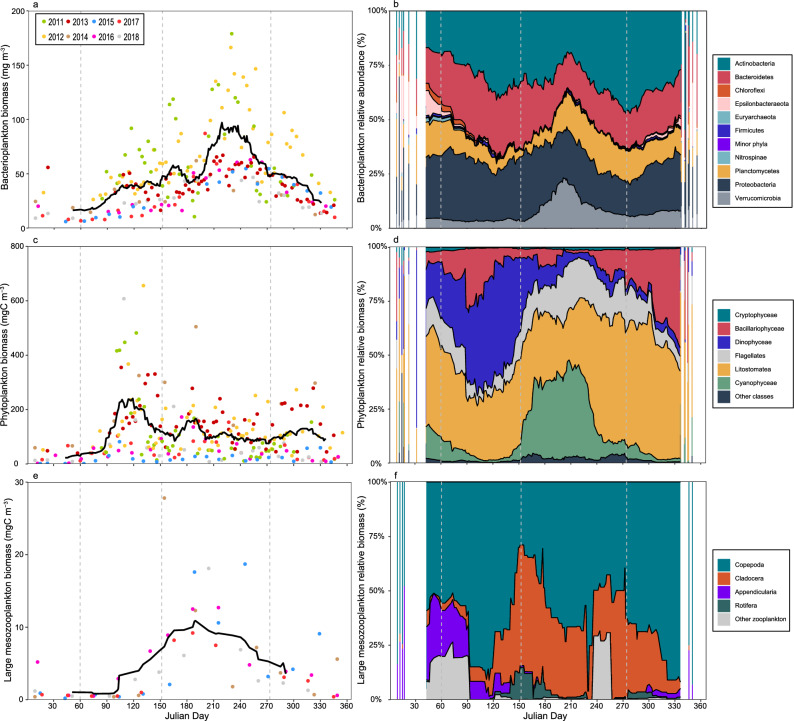
Annual trend of bacterioplankton (**a**), phytoplankton (**c**), large mesozooplankton biomass (**e**), and relative bacterioplankton (**b**) phytoplankton (**d**) and large mesozooplankton community composition (**f**). For (**b**, **d** and **f**), the ridge plot shows the moving average covering 15 julian days, combining all the years whilst bars show discrete days where the coverage is partial. Points illustrate separate samplings over the years with a different color for each year and the black lines show the moving average covering 15 julian days, combining all years. Vertical dashed lines illustrate breaks for seasons. Note that cyanobacteria community composition is included in the phytoplankton category (**d**) and not in the bacterioplankton category (**b**) in this figure (see methods).

Bacterioplankton biomass generally started to increase during early spring, reaching a plateau around 40 mg C m^−3^ until July (days 182–212) when it increased further to reach around 90 mg C m^−3^ towards the middle of the summer (Fig. 2a). During autumn, bacterioplankton biomass decreased steadily down to winter values at around 20 mg C m^−3^. The trend was present for all the years, but the bacterioplankton biomass was greater for the period 2011–2012 compared to 2013–2018. However, within the two separate periods, the interannual variability was less pronounced (Fig. 2a; Fig. S4a). Chl *a* peaked in April (days 91–120) during the spring bloom (~ 5 µg L^−1^), after which it decreased to close to pre-bloom levels (~ 1 µg L^−1^). By mid-summer (July, days 182–212)), a summer peak (~ 3 µg L^−1^) was followed by a slow decrease during autumn to winter levels (~ 0.5 µg L^−1^) (Fig. 1f). Interannual variability was low, with only two samplings (spring 2011 and 2018) having notably high values > 10 µg L^−1^ (Fig. 1f; Fig. S1f.). Phytoplankton biomass displayed a similar pattern compared to Chl *a*, with a spring bloom in April (days 91–120), reaching ~ 250 mg C m^−3^. During late spring, phytoplankton biomass had decreased to around 100 mg C m^−3^ (Fig. 2c) and a second peak was reached by mid-summer (July, 182–212) at ~ 200 mg C m^−3^. Subsequently, biomass stabilized around 100 mg C m^−3^ and decreased to winter values in December-January (25–50 mg C m^−3^). Phytoplankton were quantified using slightly different analytical methods during 2011–2014 compared to 2015–2018 (see material and methods) indicating that separate analysis of the two time periods is necessary. Biomass did not display a large interannual variability during the two periods 2011–2014 and 2015–2018 when analyzed separately. However, biomass was higher throughout the first period compared to the later (Fig. S4b). This result was probably related to the differences in the analytical procedure for the two time periods. Total biomass of large mesozooplankton displayed a seasonal trend with winter biomass of ~ 1 mg C m^−3^, followed by a rapid increase in mid-spring (May, days 121–151) peaking during early summer (June, days 152–181)) with large mesozooplankton biomass of ~ 10 mg C m^−3^ being stable for about a month (Fig. 2e). Towards the end of July (day 212), large mesozooplankton started to decrease and by autumn reached ~ 5 mg C m^−3^. The biomass of large mesozooplankton displayed low interannual variability, with similar seasonality among the years (Fig. 2e; Fig. S4c).

Analysis of 16S rRNA gene amplicons showed that Actinobacteria, Bacteroidetes, Proteobacteria were abundant along with Planctomycetes and Verrucomicrobia, and several of these phyla exhibited pronounced seasonal changes in relative abundance (Fig. 2b, Fig. 6). For example, Actinobacteria exhibited two periods of elevated relative abundance (in spring and autumn), while Verrucomicrobia peaked in summer. Bacterial community composition did not vary largely interannually but displayed recurring patterns (Fig. S5) that were similar over the study period (Fig. S8a). Microscopy analysis showed that the phytoplankton and mixotrophic communities during spring were dominated by Dinophyceae (47%), Litostomatea (ciliates, 36%), Bacillariophyceae (12%) and Flagellates (11%) (Fig. 2d, Fig. 6). During summer, Cyanophyceae (38%) and Litostomatea (35%) made up the largest proportion of the community, followed by Flagellates (23%) and Dinophyceae (11%). During autumn, Litostomatea dominated in terms of biomass, accounting for 59% of the phytoplankton community. Bacillariophyceae (20%), as well as Flagellates (16%) comprised a larger part of the community. The winter phytoplankton community had a large contribution of Litostomatea (41%), but Dinophyceae (27%), Cyanophyceae (18%) and Flagellates (11%) were also present. When investigating the periods 2011–2014 and 2015–2018 separately, phytoplankton community composition did not show a large interannual variability. However, comparing the two periods show a clear large difference (Fig. S4b; Fig. S6; Fig. S8b), related to the relative contribution of Litostomatea, Flagellates, Cryptophyceae and “Other phytoplankton” and this was probably mostly due to differences in analytical procedure and should not be interpreted as an ecological effect. In the large mesozooplankton fraction, Copepoda (63%), Cladocera (20%) and Appendicularia (16%) contributed the most to the total biomass during spring (Fig. 2f, Fig. 6, Fig. S7). Copepoda was the largest group in the community during summer (57%), when also Cladocera peaked (40%). Similarly, during autumn, Copepoda was the main group (71%), followed by Cladocera (28%) and Appendicularia (9%). Winter community’s main contributors were Copepoda (74%), Appendicularia (27%) and ‘Other zooplankton’ (21%) (Fig. 2f). The large mesozooplankton community composition showed similar recurring patterns on an annual scale (Fig. S7) and was similar over the study period (Fig. S8c). The most common bacterio-, phyto- and large mesozooplankton taxa are specified in Table S1.

**Figure 3:**
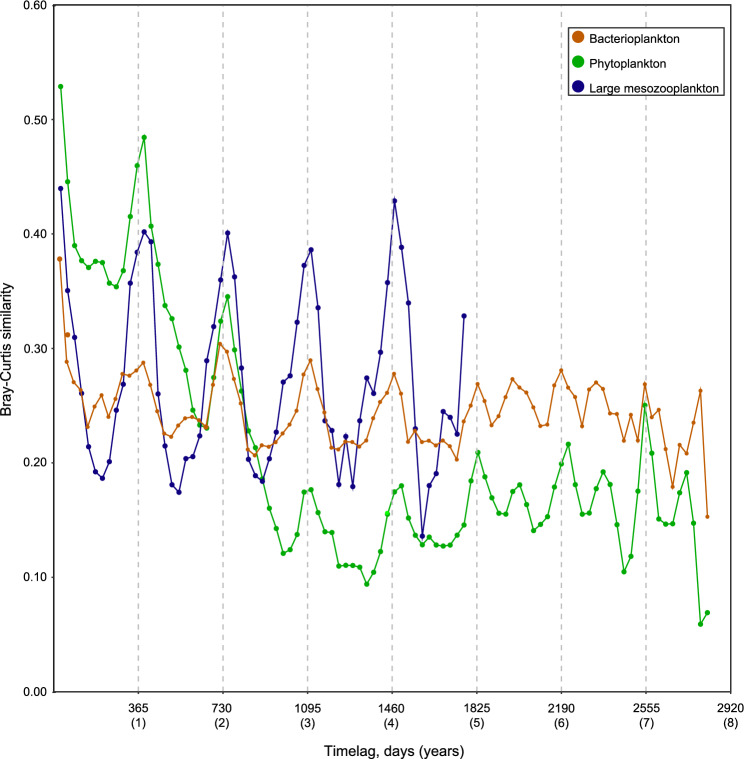
Bray–Curtis similarity plot for bacterioplankton, phytoplankton and large mesozooplankton. Dots mark monthly averages of pairwise comparisons of samplings “n” months apart. Therefore, the first point shows the average similarity of all pairs of samples collected within one month of each other, the second point for all samples collected within 2 months of each other. Vertical dashed lines illustrate breaks for years (365 days). Note that cyanobacteria community composition is only included in the phytoplankton category and not in the bacterioplankton category in this figure (see methods).

Temporal analysis of Bray–Curtis similarity values of the respective community compositions between samplings (indicative of temporal community similarity/dissimilarity) displayed a sinusoidal annual pattern for all trophic levels (Fig. 3). The seasonality pattern was more pronounced for the large mesozooplankton. Moreover, for the period when large mesozooplankton composition data was available (5 years), the dynamics in similarity values were highly coordinated with distinct peaks within only a month’s time. However, the data for large mesozooplankton was only collected once per month reducing the resolution of the seasonal cycle in these taxa. Bacterioplankton and large mesozooplankton Bray–Curtis similarity mostly varied around 0.25, whereas similarity values for the phytoplankton decreased from around 0.45 to 0.1 over periods of up to 3 years after which it stabilized (Fig. 3).

### Correlations between biotic and abiotic parameters

**Figure 4:**
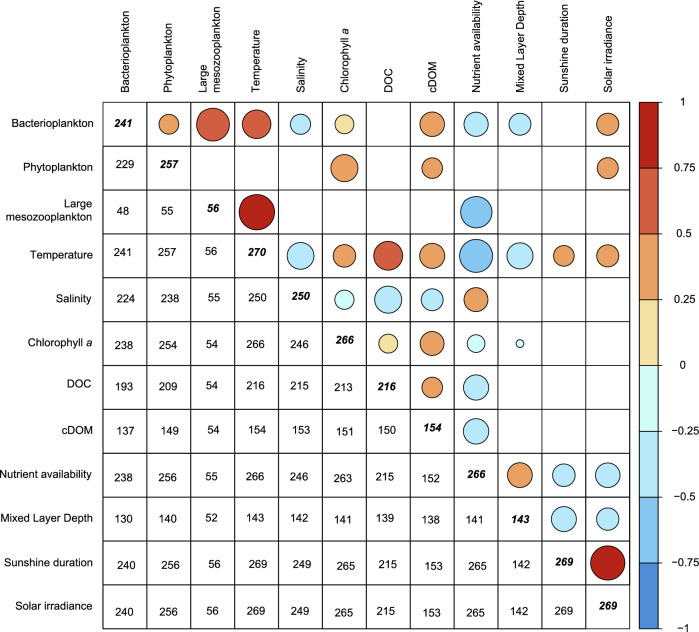
Spearman correlation matrix for biotic and abiotic parameters. Color intensity and size of circle corresponds to correlation value. Values in bold italics in diagonal show the total number of datapoints available for each parameter. Values below diagonal display number of pairwise comparisons for the separate correlations. Blank squares show when the correlation significance level was *p *> 0.05.

Bacterioplankton biomass displayed significant correlations with most of the investigated variables, except for DOC and sunshine duration (Fig. 4). Bacterioplankton biomass showed positive correlations with phytoplankton biomass, large mesozooplankton biomass, temperature, Chl *a*, cDOM and solar irradiance whilst the relationship was significantly negative with salinity, nutrient availability, and mixed layer depth. Phytoplankton biomass only displayed positive correlations with bacterioplankton biomass, Chl *a*, cDOM, and solar irradiance. Mesozooplankton biomass was positively correlated with bacterioplankton biomass and temperature and showed negative correlation with nutrient availability. Moreover, as could be expected, the abiotic variables showed multiple correlative relationships, among which temperature and nutrient availability displayed the largest number of correlations. Both temperature and nutrient availability displayed significant relationships with all investigated variables, except for phytoplankton biomass (Fig. 4).

### Co-occurrence in the plankton food web

**Figure 5:**
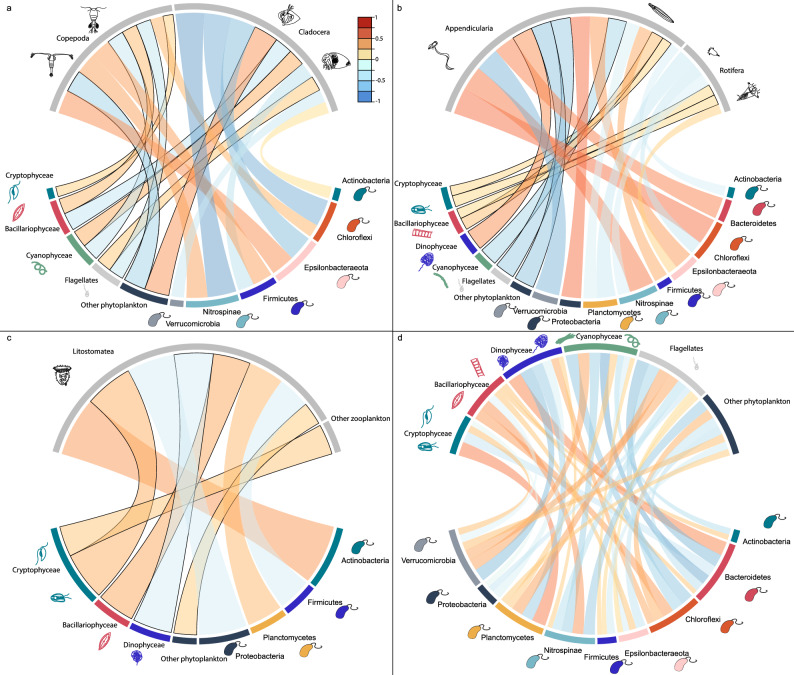
Chord diagram visualizing Spearman correlations of the relative biomass among predators (large mesozooplankton and Litostomatea) and prey (phyto- and bacterioplankton). Copepoda and Cladocera (**a**), Appendicularia and Rotifera (**b**), Litostomatea and ‘Other zooplankton’ (**c**) versus prey item as well as correlations between phytoplankton and bacterioplankton (**d**). Width and color of links are proportional to correlation coefficient. Only significant (*p *< 0.05) correlations are displayed. Links among predators and phytoplankton are presented with black border for distinction.

Relative biomasses of bacterio-, phyto- and large mesozooplankton were used to infer potential interactions in the planktonic food web (Fig. 5), although one should note that correlations could also appear without any direct or indirect interaction. Here, we considered large mesozooplankton and Litostomatea (predominantly the mixotrophic ciliate *Mesodinium rubrum*) as predators whilst bacterioplankton and phytoplankton groups were prey items. The relative biomass of Copepoda was significantly correlated with the relative biomasses for five out of the six phytoplankton groups included, as well as four out of the 11 bacterioplankton groups (Fig. 5a). Strongest correlations were obtained with Cyanophyceae and ‘Other phytoplankton’ as well as Epsilonbacteraeota. Cladocera displayed significant correlations with four phytoplankton groups, and six bacterioplankton groups. The relative biomass of Rotifera was negatively correlated with two phytoplankton groups, and four bacterioplankton groups (Fig. 5b). Appendicularia had the highest number of significant correlations, with all phytoplankton groups, and nine bacterioplankton groups. Litostomatea (ciliates) displayed significant correlations with four phytoplankton groups, and three bacterioplankton groups (Fig. 5c). ‘Other zooplankton’ only displayed one significant correlation (Cryptophyceae). From the bacterioplankton community, relative biomasses of Euryarchaeota did not show any significant correlations with predators. Note that some care should be applied when interpreting the correlations with phytoplankton since they were estimated using slightly different analytical methods during 2011–2014 compared to 2015–2018. Furthermore, *Mesodinium rubrum* also has an autotropic life style^74,75^ and the correlations found here should be evaluated in light of the mixotrohic capacity of this species.

Next, we determined potential interactions between organisms in the microbial food web that compete for resources such as nutrients and carbon sources and in some cases light for energy production. Main phytoplankton groups in spring, such as Dinophyceae, showed a strong positive correlation (co-occurrence) with the bacteria Bacteroidetes and negative correlations (different seasonal pattern) with Verrucomicrobia and Planctomycetes (Fig. 5d). Additionally, the most common phytoplankton in summer, Cyanophyceae, had positive correlations with Verrucomicrobia and Planctomycetes and Firmicutes but negative correlations with the remaining groups excluding Euryarchaeota and ‘Minor phyla’.

### Seasonal carbon pool dynamics

**Figure 6:**
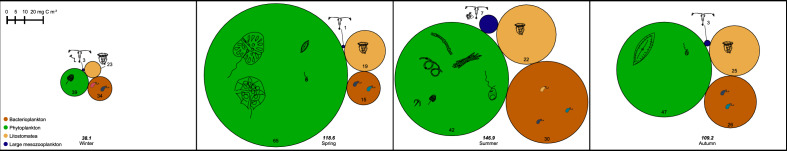
Seasonal carbon content in various plankton groups. Only samplings when all trophic levels were available are included (n = 46). The size of circles corresponds to carbon content (mg C m^−3^), and relative contribution to the total carbon pool is presented inside or above each circle. Values in bold italics are seasonal total carbon content for the different compartments of the planktonic food web studied here (i.e. bacterioplankton, phytoplankton, Litosomatea (at 2 m) and large mesozooplankton from water column from 30 m up to surface)). Drawings illustrate the major taxa in each plankton group and its size illustrate relative contribution and correspond to drawings in Fig. 5. Data presented in Table S2.

When quantifying the carbon content in various plankton groups in different seasons, both inter- and intra-seasonal dynamics were present. Bacterioplankton carbon was highest during summer, but as the total carbon content also peaked during summer, the relative contribution from bacterioplankton during summer was 30% of seston carbon content (Fig. 6; Table S2). The relative contribution from bacterioplankton peaked during winter (34%), even though the amount of carbon was the lowest. Bacterioplankton relative contribution to the total carbon in the plankton food web was lowest during spring, and during autumn bacterioplankton accounted for 25% of the total carbon (Fig. 6; Table S2). Phytoplankton consistently accounted for > 39% of the total carbon in the plankton food web. During spring, phytoplankton carbon and its relative contribution was highest (65%), whilst reaching minimal values in winter (39%). During summer and autumn, phytoplankton carbon was at a similar level, considering both amount and relative contribution (Fig. 6; Table S2). Litostomatea (exclusively the ciliate *M. rubrum*) made a noteworthy contribution to total carbon in the plankton communities, comprising > 20% of the total carbon throughout all seasons. During winter, Litostomatea had the lowest carbon levels, whilst the highest levels were found during summer. For most of the seasons, large mesozooplankton carbon and relative contribution was low (1–3%), except during summer when values peaked (7%). During winter and spring, large mesozooplankton carbon was at its lowest. Autumn large mesozooplankton carbon was two times higher than winter and spring levels (Fig. 6; Table S2).

## Discussion

This multi trophic-level and multi-year study showed seasonal patterns in all trophic levels and a strong connectedness between abiotic parameters, especially with temperature and nutrient availability. Furthermore, in the plankton communities, potential interactions were identified among groups in all trophic levels. These data can be used in a continued work to understand food web structure and interactions of bacterio-, phyto- and large mesozooplankton communities and to provide new knowledge on the interdependencies of trophic levels at the crossroads of the microbial and traditional food webs. In our study, focusing on bacterio-, phyto- and large mesozooplankton, combining biomass, community composition and co-occurrences, an extra noteworthy observation was that phytoplankton made up most of the plankton carbon biomass throughout all seasons. However, it should be emphasized that there are also other compartments of the microbial food web which are not included here which can contribute a significant amount of carbon to the system, e.g. picophytoplankton, viruses, fungi, archaea and smaller zooplankton. When investigating temporal community similarity some striking features were revealed; as expected, but rarely shown, Bray–Curtis similarity was coordinated over time and among all plankton groups studied. This is in line with other studies on specific taxa of plankton suggesting similar patterns of recurring community similarity^8–10,78,79^.This implies that whilst the community composition changes over the seasons, it returns to a stable state over a year. This is a prerequisite to create biomass which was, relatively stable over the seasons, except in winter when the planktonic carbon pool was at its lowest.

Temperature has been reported to alter bacterial community composition^80,81^, as well as having a positive relationship with bacterial biomass and production^34,40,82,83^. In the present study, bacterioplankton biomass peaked just after sea surface temperature had reached its maximum and the two parameters were strongly correlated. Phytoplankton biomass did not display any correlation with temperature, in contrast to the unimodal relationship described by Legrand, et al.^34^ as well as the positive relationship with filamentous cyanobacteria^84^ and other phytoplankton phyla^40^. Zooplankton biomass shows a strong positive correlation with temperature^85^. In a recent study, mixotrophic and autotrophic biomass was found to correlate positively with temperature, whereas the biomass of heterotrophs did not show this relationship^86^. This illustrates that while being a largely influential factor, temperature is not the sole controlling parameter for plankton communities in the Baltic Sea. The interannual and within-year variation in temperature, mixed layer depth, salinity, and nutrient availability also illustrates that the sampled station is dynamic regarding the abiotic conditions.

Nutrient availability was inversely correlated to several abiotic variables as well as to bacterio- and large mesozooplankton biomass. Phytoplankton biomass was not correlated to nutrient availability, which is opposite to previous findings, e.g.^87^. This discrepancy could be due to the bimodal phytoplankton dynamics in the Baltic Proper, having one spring bloom comprised of Dinophyceae, Litostomatea and Bacillariophyceae followed by a summer bloom dominated by Cyanophyceae and Litostomatea. For example, decoupling between nutrient levels and cyanobacterial abundances has previously been reported^84,88^. Filamentous, heterocystous N_2_ fixers dominated the summer bloom, and these could fuel the system with bioavailable nitrogen for hetero- and autotrophic picoplankton^89^, as well as grazers through ingestion and other phytoplankton and microbes by exudates^90^.

Bacterioplankton community composition displayed a seasonal pattern, and the Bray–Curtis similarity was stable over the years. This pattern indicate that the composition of the communities follow a seasonal pattern and that the communities are more similar during the same season (even when several years apart), but the community composition is most different when comparing opposite seasons (~ 6 months apart)^91^. A high Bray–Curtis similarity over time implies temporal stability, concurrently a low similarity implies changes in the communities^91^. The dominance of filamentous *Aphanizomenon flos-aquae* and *Nodularia spumigena* in the microscopy counts during summer was consistent with previous reports from the Baltic Proper^34,69,84,92,93^. The phytoplankton and mixotrophic community showed a pronounced seasonality, with Litostomatea, Dinophyceae, Bacillariophyceae, and Cyanophyceae all being major contributors at different time points on an annual scale, which has been shown partially previously^28,69^. A change in the spring community composition from a pelagic system where diatoms dominated spring and autumn blooms, and cyanobacteria and green algae prevailed during summer blooms, to a revised picture with Dinophyceae, rather than Bacillariophyceae, being the dominant phytoplankton in spring surface waters has been reported previously^34,69,93,94^ and was also true for the present study. Although the large mesozooplankton community was sampled at a lower frequency than bacterio- and phytoplankton, data covering five years still allowed for exploring the community composition. The common paradigm in zooplankton ecology is that Copepoda is the dominating large mesozooplankton taxa in the Baltic Sea^35,95^. Copepoda were indeed the major taxa in the large mesozooplankton community during all seasons, consistently accounting for > 55% of the community (average per season). Yet, other taxa such as Appendicularia appear to have a significant role in the Baltic Sea in relation to copepods and cladocerans. Small-sized zooplankton were not included in this study, due to the sampling technique, making inferences about them in terms of abundance and biomass in relation to large mesozooplankton impossible. However, other studies have shown that small-sized zooplankton, such as rotifers and small cladocerans, play a critical role, both in terms of biomass and abundance, in the Baltic food web^8,10^. Furthermore, ciliates (Litostomatea; *M. rubrum*) also contributed to a large fraction of the carbon pool throughout the year, although some studies suggest that their life-style is dominated by autotrophy^74,75^, questioning their importance as predators. This suggests that a diverse and dynamic plankton community should be considered when assessing the food web structure, which could help explain the interactions in the planktonic realm.

Bacterioplankton community composition was consistent over the study period and displayed recurring seasonality throughout the 8 years. Bacterioplankton communities were more similar when comparing seasons among years, than when comparing different seasons within a year. The largest differences in seasonal community composition were between summer and winter and was related to the relative contribution of Nitrospinae, Chloroflexi, Epsilonbacteraeota and Minor phyla during winter. In contrast, phytoplankton community composition displayed two periods of varying community composition. Analytical methods for microscopic phytoplankton counts were different in 2011–2014 and 2015–2018, respectively. Hence, patterns over time are not relevant to study. Within the separate periods (2011–2014 and 2015–2018), phytoplankton community showed a low variability, where relative contribution of Cyanophyceae and Dinophyceae had the largest impact on the seasonal distinction of the community. Mesozooplankton community composition was similar over the years of the study and was dominated by Copepoda throughout. Relative increases of Cladocera and Rotifera during summer in conjunction with relative increases of Appendicularia during winter had an impact on the community composition over seasons, but not among years.

The strong seasonality observed in the phyto- and large mesozooplankton communities indicated seasonally reoccurring patterns in community structures and that the turnover was similar for all investigated plankton communities. Recently, Yeh and Fuhrman^32^ found different diversity patterns for protists and bacterioplankton at the Californian coast with more pronounced changes in eukaryotic communities, using amplicon sequencing. In contrast, our microscopic counts indicated a strong seasonality in both phytoplankton and large mesozooplankton communities compared to bacterioplankton. When combining all trophic levels, carbon biomass was highest during summer, followed by spring, autumn, and lowest during winter, similar to findings from lakes and river plumes in temperate regions^26,27^. Also, the relative contribution from various plankton groups matched previous reports^26,27^, where phytoplankton contributed most to the planktonic total carbon pool, followed by bacterioplankton and Litostomatea, whilst large mesozooplankton only had a minor contribution to the carbon pool.

Interactions between organisms include e.g., grazing, symbiosis, competition for resources, and parasitism. As such, a negative correlation can be interpreted in several ways, including a strong top-down effect (predation) or occurrence in different seasons (e.g., spring or summer adapted taxa), alternatively organisms may occupy the habitat during different periods of the year. If predators and prey are influenced by similar environmental drivers without strong top-down effects, they will co-occur (positive correlation). Grazing might result in the release of substrates (sloppy feeding) which provides new resources for organisms, and bulk plankton analyses might also capture pathogenic microbiomes, which will lead to positive correlations between species. Also, trophic effects are possible e.g., when macro- and mesozooplankton graze on microzooplankton and thus release grazing pressure on other planktonic groups (positive and negative correlations of biomass between separate groups). However, one should always keep in mind that significant correlations could arise without any interaction between the organisms whatsoever. Hence, correlation analyses cannot offer a definitive answer on effects and causation, but it enables investigations for potential links. A general caveat in this study is also that small sized zooplankton are not included in the sampling which may underestimate the correlations between primary producers and consumers.

In our study, Copepoda correlated mainly negatively with various phytoplankton taxa, possibly due to grazing. However, phytoplankton which are not vulnerable to grazing (for instance due to morphology) could be negatively correlated to Copepoda not only due to grazing but due to increased competition over resources from other phytoplankton or bacterial species which are positively correlated with Copepoda, e.g., Cyanophyceae and Flagellates. Copepoda correlated mainly positively with various bacterioplankton taxa, which could be the result of sloppy feeding. However, it should be noted that zooplankton were sampled in the top 30 m of the sampling station whereas bacterio- and phytoplankton were sampled at two meters. Zooplankton are known to have both seasonal and diel habitat partitioning. For example, many cladocera are mostly abundant in summer in warm surface water, whereas many appendicularians predominantly avoid the warm surface water^8–10^. Hence, some zooplankton taxa in our data set may have a different niche compared to the bacterio- and phytoplankton we sampled in surface water (2 m), and the correlations observed may not be a function of true direct or indirect interactions.

Furthermore, zooplankton have been shown to interact with bacterioplankton in more ways than predation, both by attracting and enabling growth in the zoosphere, as well as farming them inside or on the outside of the body^97^. In the present study, relative biomass of Litostomatea had no significant correlations with other predators. Ciliates have been reported to forage successfully on phytoplankton, occasionally at rates more than double that of zooplankton^98^. Litostomatea was negatively correlated with Dinophyceae (predation and/or competition) and positively correlated with Cryptophyceae, Bacillariophyceae and ‘Other phytoplankton’ and showed mainly positive relationships with bacterioplankton, suggesting that Litostomatea does not exert a substantial feeding pressure on phytoplankton and bacterioplankton.

Relationships among phyto- and bacterioplankton have been studied in several systems and experiments, but studies covering longer time periods and with information on community composition are sparse^99–103^. However, Yeh and Fuhrman^32^ recently described how prokaryotic and protist communities differ in diversity over both depths and time. Considering the current findings and previous knowledge, plankton biomass and community composition is associated to shifts in e.g., temperature and nutrient availability. In the present study, several positive and negative correlations among phyto- and bacterioplankton were present, further suggesting functional couplings, some potentially being top-down control and some bottom-up control. Previous studies have found that Alphaproteobacteria and Bacteroidetes were associated to phytoplankton spring blooms, whereas the summer bloom was more associated with Alpha- and Gammaproteobacteria and Bacteroidetes^104–106^. Also, filamentous cyanobacteria had tight couplings to Bacteroidetes, Gammaproteobacteria and Verrucomicrobia^107^, which is in line with the finding in our study. Furthermore, species richness and diversity of particle-attached and free-living bacteria have been shown to have varying responses to phytoplankton blooms^108^. This shows the connectivity among trophic levels but also the complexity when interpreting multi-level associations. In the broader context there have been large changes occurring in the Baltic Sea during the twentieth century. Anthropogenic pressures such as climate change, eutrophication, and over-exploitation, together with climate variability, have led to regime shifts^4,109^. Recent studies suggest that the Central Baltic Sea is in a pelagic dominated, high productivity state with, e.g., low abundances of cod and copepods such as Pseudocalanus and high phytoplankton biomass during summer^110^. Hence, this dataset should be interpreted in this broader context and collection of time series data is important to detect future possible regime shifts.

## Conclusion

This study provides insights into the structure and seasonal dynamics of different trophic levels of the pelagic microbial food web and how the trophic levels correlate with each other and environmental drivers. At present, studies covering several years, seasons, trophic levels, and environmental drivers are scarce. As such, our study offers a rare window into seasonal succession, food web structure, and interactions of bacterio-, phyto-, and large mesozooplankton communities. Our results showed a clear and stable seasonal succession of the investigated plankton communities. For phyto- and large mesozooplankton there was a pronounced reoccurring pattern with highest similarity every 12 months. Also, we found a strong interconnectivity between bacterioplankton and other parts of the aquatic environment. Temperature and nutrient availability correlated with all variables except for phytoplankton biomass, illustrating that these two environmental drivers are crucial for shaping the pelagic food web. Plankton community composition displayed a low interannual variability and the different seasons within a year were more dissimilar than matching seasons among years.

This study contributes a baseline of the food web structure in the plankton realm and here Dinophyceae, not Bacillariophyceae, dominated the spring phytoplankton bloom in the Baltic Proper between 2011 and 2018. Another striking result is that Litostomatea (ciliates) and Appendicularia contribute to the food web in such a large extent. Important members in the phytoplankton and bacterioplankton community were Cyanophyceae, Actinobacteria, Bacteroidetes, and Proteobacteria. Considering the recognition of the importance of interspecies interactions for community dynamics and biogeochemical processes^111–113^, time series research should include all trophic levels for a holistic understanding of the various food web interactions. Future studies should continue to determine the nature of the interactions using more mechanistic studies to determine the nature of direct predation, competition and mutualistic effects as well as including understudied groups such as picophytoplankton, viruses, fungi, archaea and microzooplankton.

## Supplementary Information

Supplementary Information.

## Data Availability

Data will be available upon request. 16S data are deposited in the EMBL-EBI European Nucleotide Archive repository (https://www.ebi.ac.uk/ena), accession numbers PRJEB42455, SRP048666, PRJEB52855, PRJEB52782, PRJEB52780, PRJEB52772, PRJEB52627, PRJEB52496, PRJEB52828, PRJEB52837 and PRJEB52854.
