# Supplementary material for: Multiyear analysis uncovers coordinated seasonality in stocks and composition of the planktonic food web in the Baltic Sea proper
Source: Sci Rep. 2023 Jul 22;13:11865. doi: 10.1038/s41598-023-38816-0 (PMC10363133; doi:10.1038/s41598-023-38816-0)
Supplement: Supplementary file 1 — Supplementary Information. [file 41598_2023_38816_MOESM1_ESM.docx]

Multiyear analysis uncovers coordinated seasonality in stocks and composition of the planktonic food web in the Baltic Sea Proper

**Emil Fridolfsson^1^, Carina Bunse^1,2^, Elin Lindehoff^1^, Hanna Farnelid^1^, Benjamin Pontiller^1,3^, Kristofer Bergström^1^, Jarone Pinhassi^1^*, Catherine Legrand^1,4^*, Samuel Hylander^1^***

# **Supplementary material**


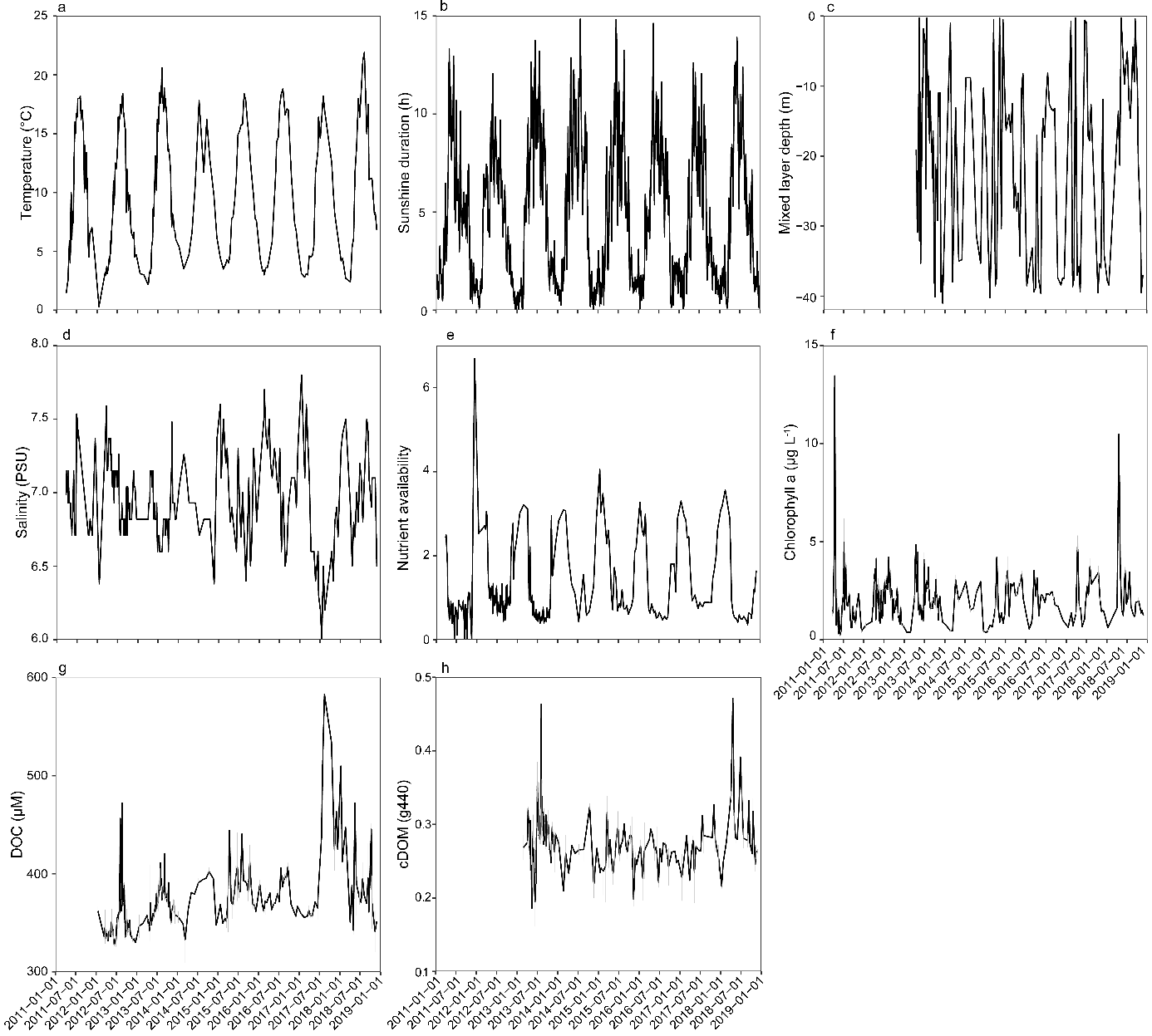


**Figure S1.** Temporal variation of sea-surface temperature (a), sunshine duration (b) mixed layer depth (sigma theta 0.125) (c), salinity (d), nutrient availability (e), Chlorophyll *a* concentration (f), levels of dissolved organic carbon, DOC (g), and colored dissolved organic matter, cDOM (h), spanning the entire study period. Grey bars illustrate standard deviation for technical replicates were applicable.


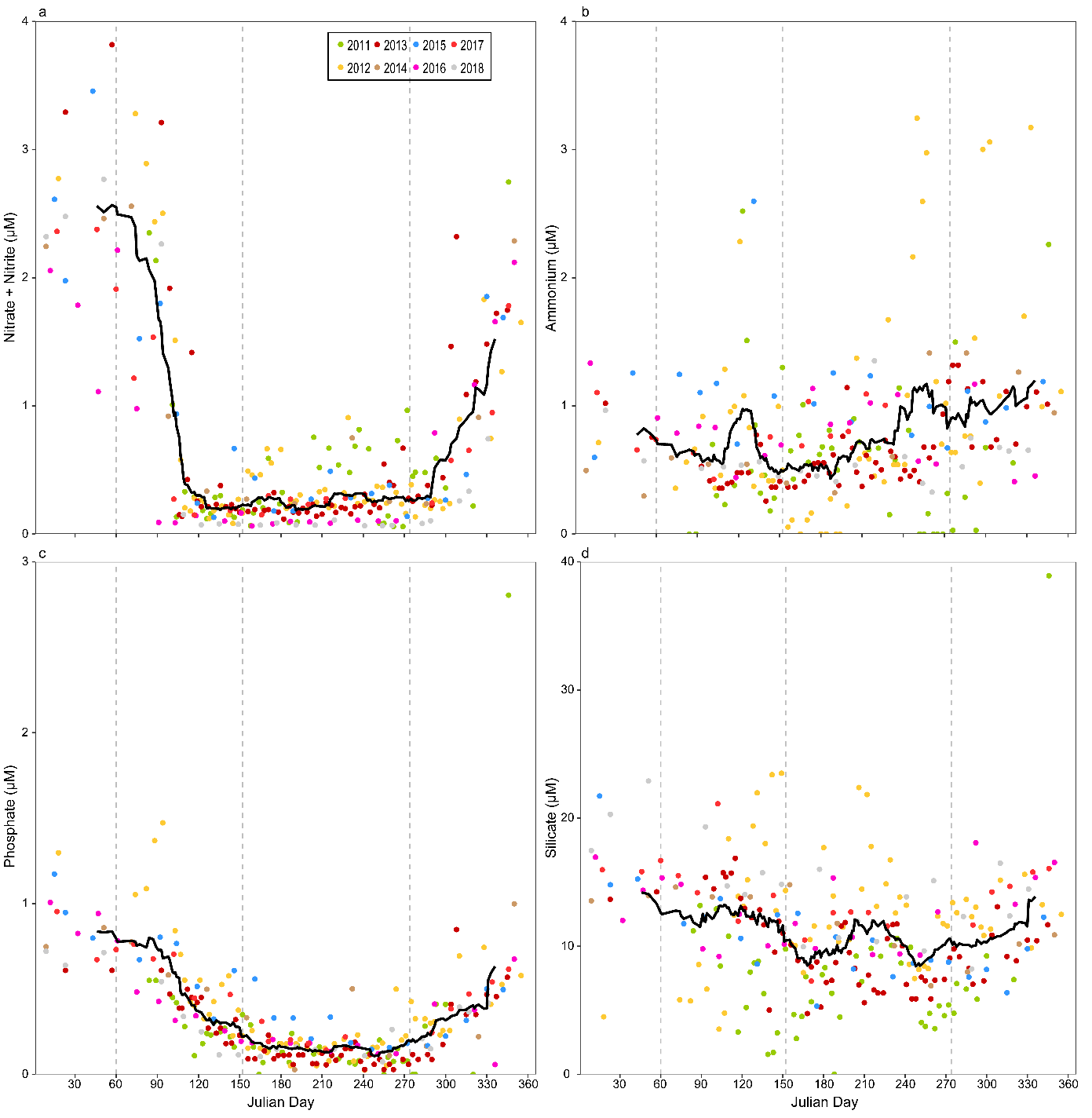


**Figure S2.** Annual variation of nitrate + nitrite (a), ammonium (b), phosphate (c) and silicate (d) concentrations_._ Points illustrate separate samplings over the years and lines show the moving average covering 15 Julian days, combining all the years.

**
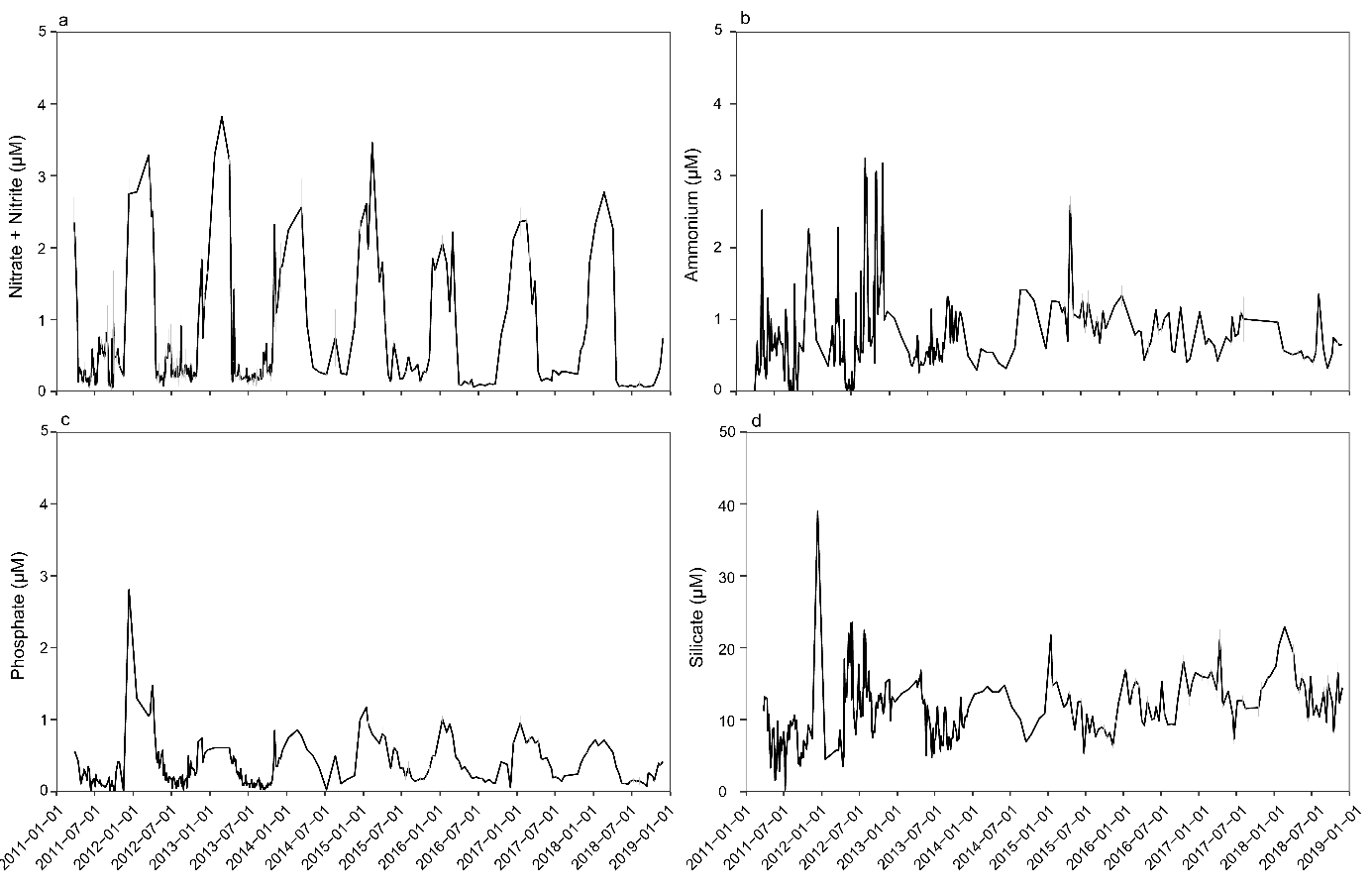
**

**Figure S3.** Temporal variation of nitrate + nitrite (a), ammonium (b), phosphate (c) and silicate (d) concentrations_._ Grey bars illustrate standard deviation for technical replicates were applicable.


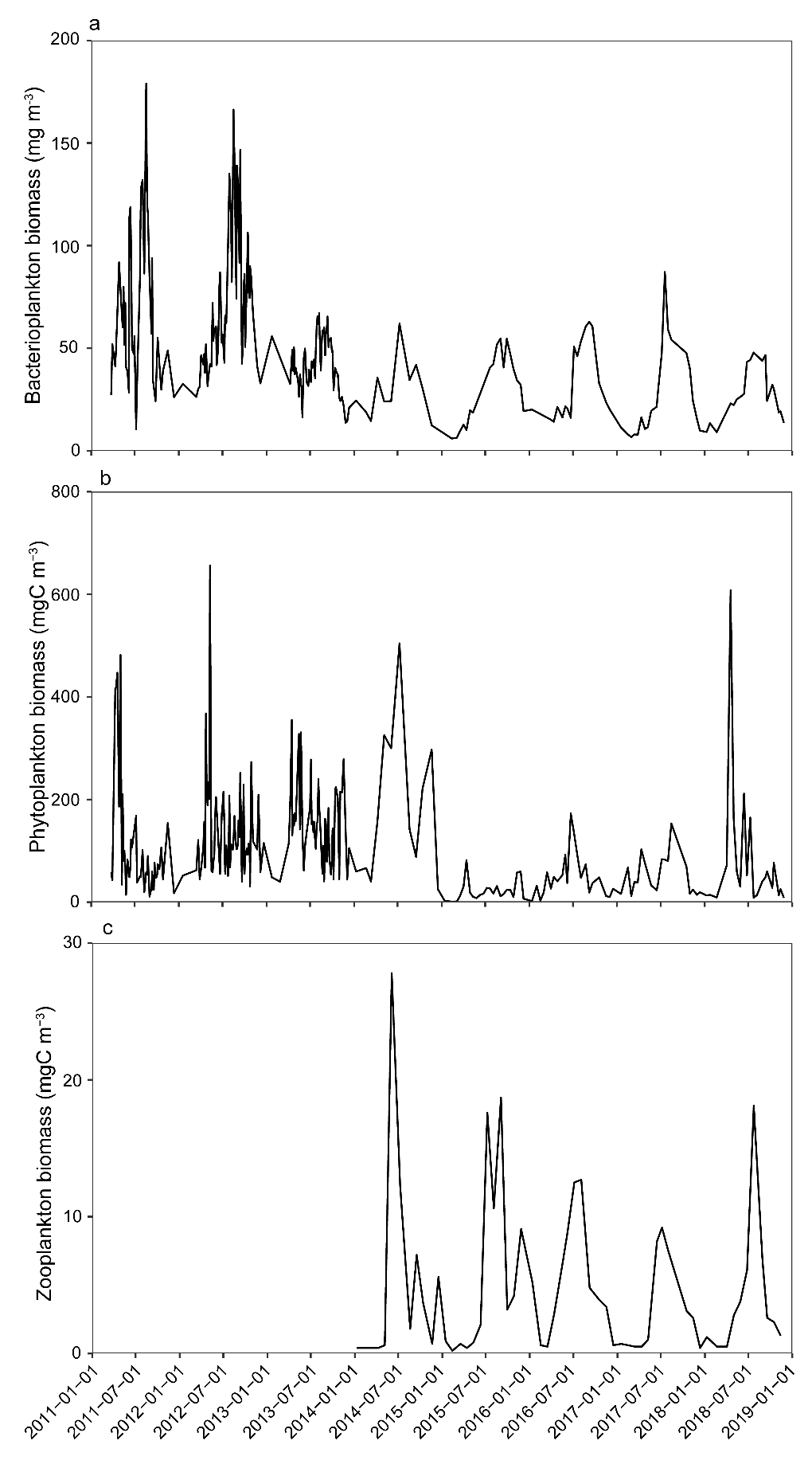


**Figure S4.** Temporal variation of bacterioplankton (a), phytoplankton (b), and large mesozooplankton biomass (c). Note that phytoplankton microscopy methods were different during 2011-2014 compared to 2015-2018.

**
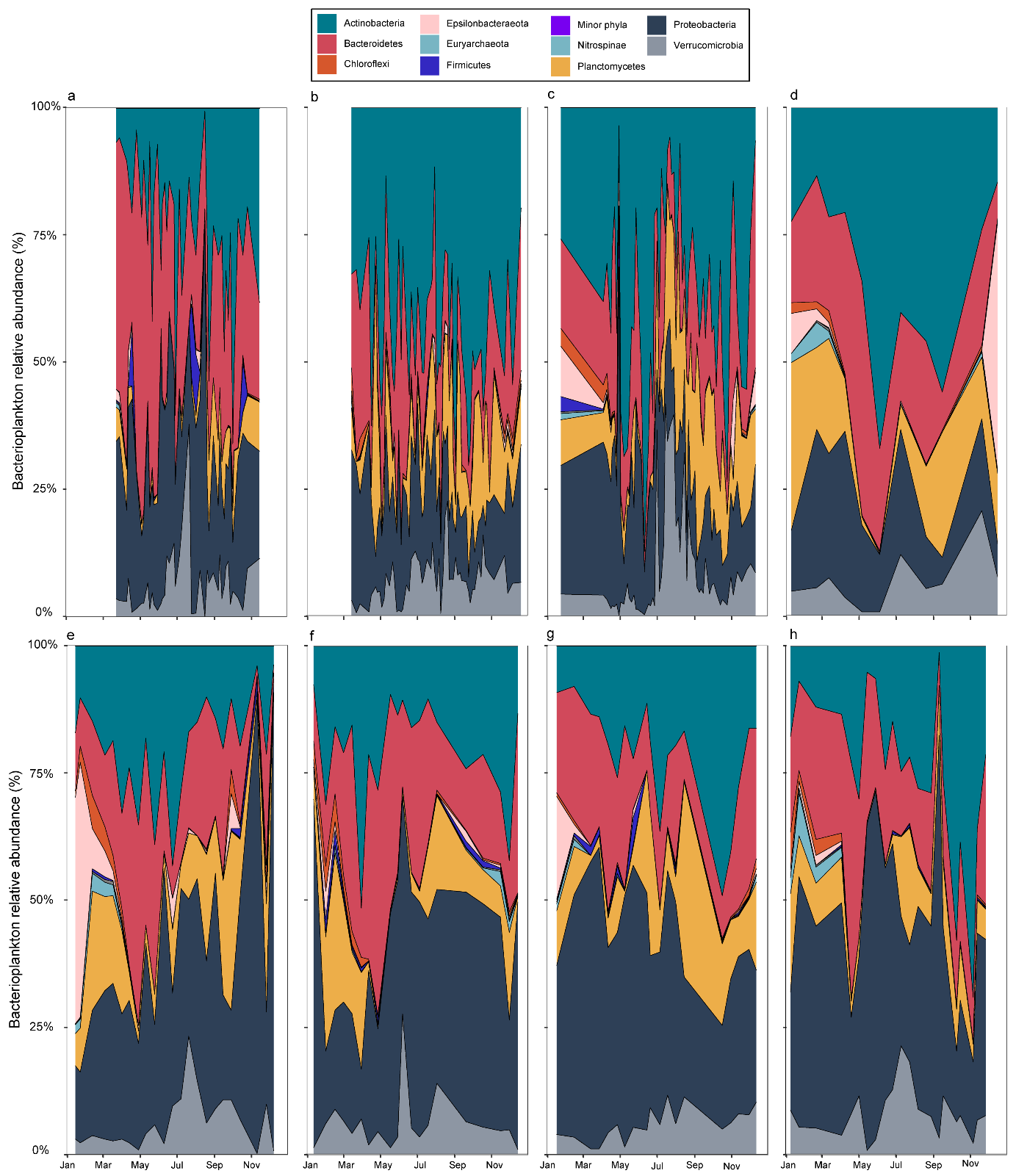
**

**Figure S5**. Temporal variation of bacterioplankton relative abundance for the separate years. 2011-2014 is presented in the top row (a-d) and 2015-2018 is presented in the bottom row (e-h).


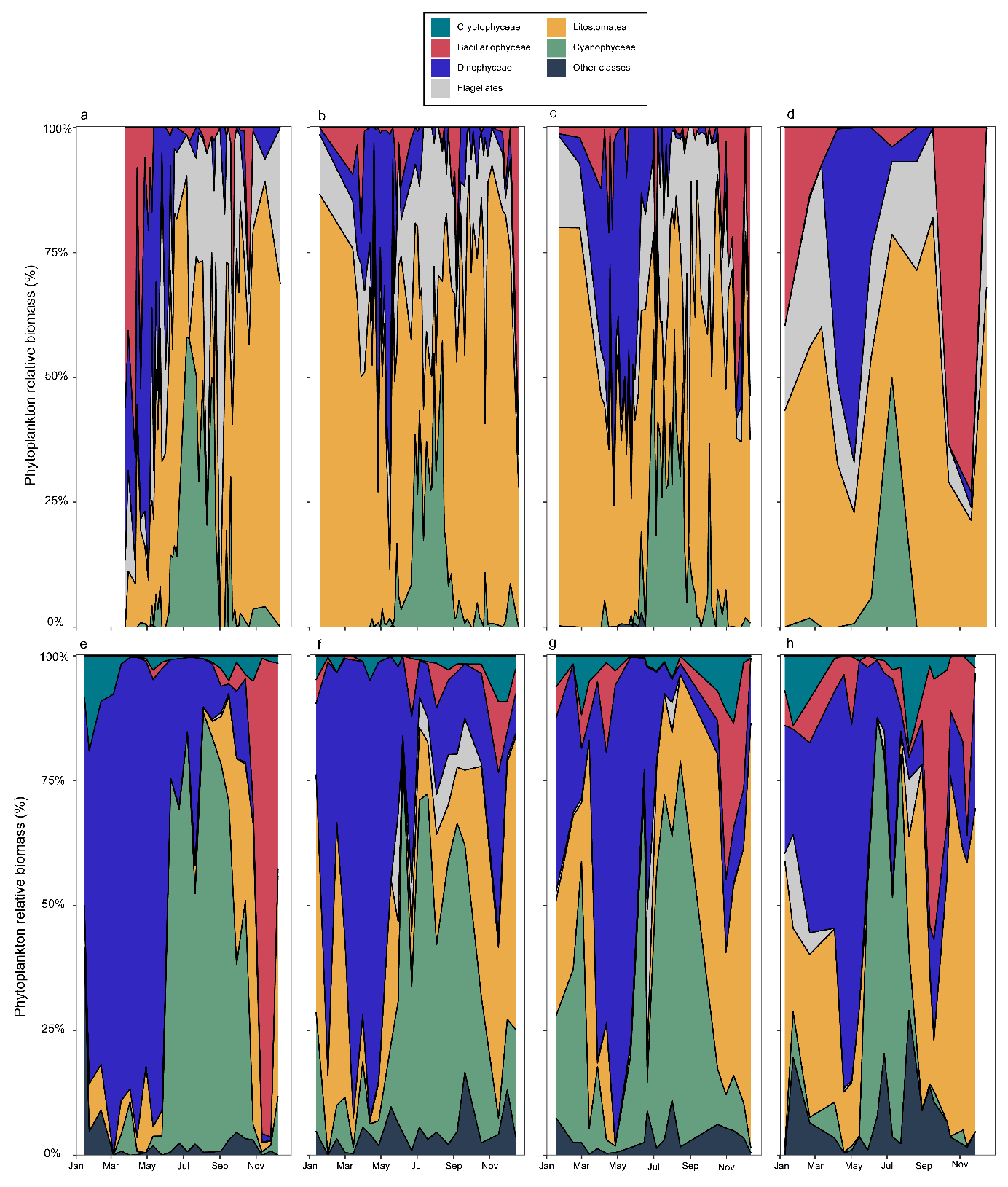


**Figure S6**. Temporal variation of phytoplankton relative biomass for the separate years. 2011-2014 is presented in the top row (a-d) and 2015-2018 is presented in the bottom row (e-h). Note that phytoplankton microscopy methods were different during 2011-2014 compared to 2015-2018.

**
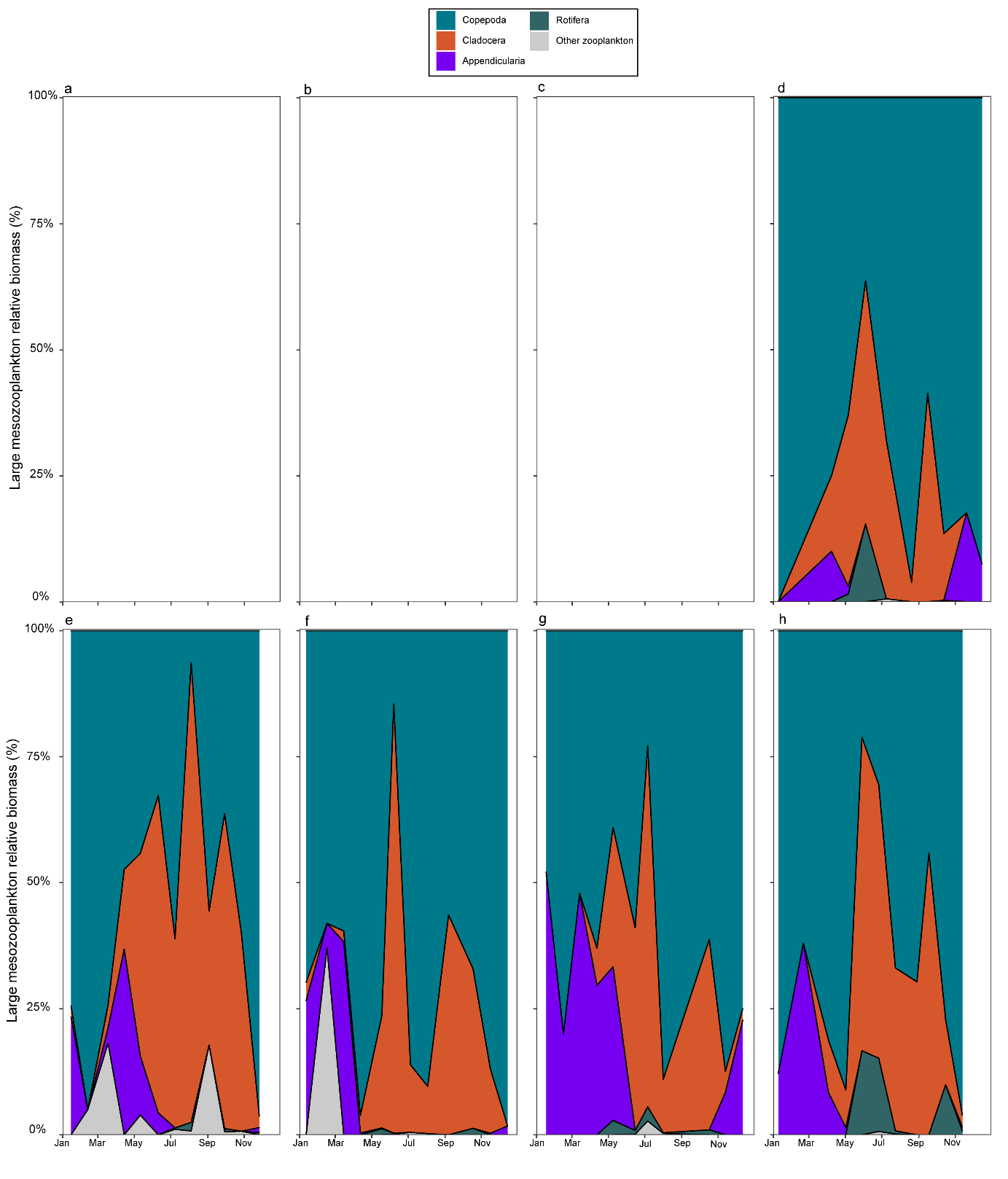
**

**Figure S7**. Temporal variation of zooplankton relative biomass for the separate years. 2011-2014 is presented in the top row (a-d) and 2015-2018 is presented in the bottom row (e-h). Zooplankton data available from 2014.

**
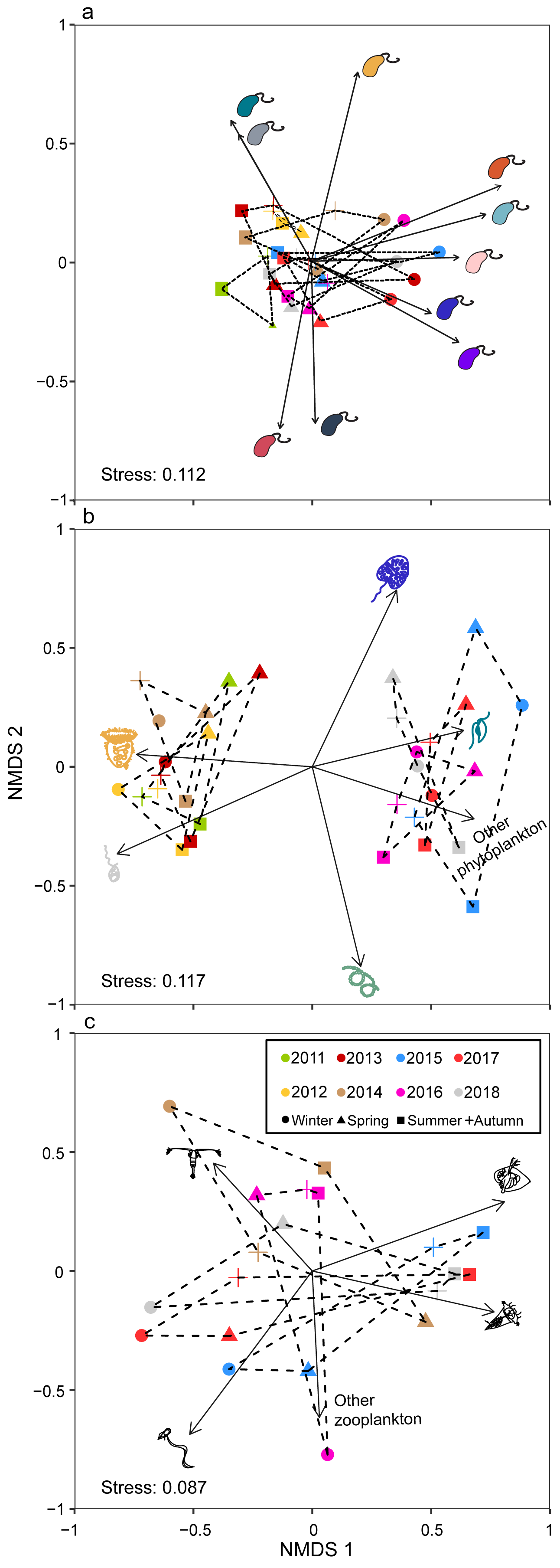
**

**Figure S8**. Non-metric multidimensional scaling (NMDS) plot of community composition of bacterioplankton (a), phytoplankton (b), and zooplankton (c). Each symbol represents median community composition (Bray-Curtis dissimilarity) for each separate year and season whilst color represents year. Dashed lines connect seasons within a year. Arrows and drawings illustrate fitted vectors and correspond to drawings in Fig. 5. Note that phytoplankton microscopy methods were different during 2011-2014 compared to 2015-2018.

**Table S1.** Top 10 taxa of bacterioplankton (family, including cyanobacterial taxa), phytoplankton (genus/species) and zooplankton (genus/species). Ordered according to relative abundance or biomass.

| Season | Bacteria | Phytoplankton | | Zooplankton | |
| --- | --- | --- | --- | --- | --- |
|  | Abundance | Abundance | Biomass | Abundance | Biomass |
| Winter | *Flavobacteriaceae* | *Flagellates* | *Mesodinium rubrum* | *Appendicularia* | *Appendicularia* |
|  | *Thiovulaceae* | *Binuclearia lauterbornii* | *Peridiniales* sp*.* | *Acartia* sp*.* | *Chaetognatha* |
|  | Unknown | *Teleaulax amphioxeia* | *Dinophysis norvegica* | *Centropages* sp*.* | *Acartia* sp*.* |
|  | *Cyanobiaceae* | *Plagioselmis prolonga* | *Thalassiosira* sp*.* | *Fritillaria* sp*.* | *Centropages hamatus* |
|  | *Rhodobacteraceae* | Unicell | *Binuclearia lauterbornii* | *Acartia longiremis* | *Fritillaria* sp*.* |
|  | *Ilumatobacteraceae* | *Chaetoceros* sp*.* | *Aphanizomenon flos-aquae* | *Centropages hamatus* | *Temora longicornis* |
|  | NS9 marine group | *Peridiniales* sp*.* | *Dinophysis acuminata* | *Temora longicornis* | *Acartia longiremis* |
|  | *Pseudomonadaceae* | *Heterocapsa triquetra* | *Teleaulax amphioxeia* | *Pseudocalanus* sp*.* | *Centropages* sp*.* |
|  | *Rubinisphaeraceae* | *Chrysochromulina* sp*.* | *Flagellates* | *Bosmina* sp*.* | *Pseudocalanus* sp*.* |
|  | *Burkholderiaceae* | *Leucocryptos marina* | *Gyrodinium* sp*.* | *Gastropoda* | *Pseudocalanus acuspes* |
| Spring | *Flavobacteriaceae* | *Flagellates* | *Mesodinium rubrum* | *Appendicularia* | *Evadne normanni* |
|  | *Cyanobiaceae* | *Chrysochromulina* sp*.* | *Scrippsiella* sp*.* | *Acartia* sp*.* | *Appendicularia* |
|  | *Ilumatobacteraceae* | *Chaetoceros* sp*.* | *Protoperidinium* sp*.* | *Synchaeta* sp*.* | *Acartia tonsa* |
|  | *Rhodobacteraceae* | *Teleaulax amphioxeia* | *Flagellates* | *Evadne nordmanni* | *Acartia* sp*.* |
|  | *Microbacteriaceae* | *Scrippsiella* sp*.* | *Peridiniella catenata* | *Fritillaria* sp*.* | *Evadne* sp*.* |
|  | *Burkholderiaceae* | *Aphanizomenon flos-aquae* | *Aphanizomenon flos-aquae* | *Acartia tonsa* | *Acartia bifilosa* |
|  | *Sporichthyaceae* | *Achnanthes* sp*.* | *Dinophysis acuminata* | *Centropages* sp*.* | *Fritillaria* sp*.* |
|  | Unknown | *Heterocapsa* sp*.* | *Scrippsiella hangoei* | *Oikopleura* sp*.* | *Oikopleura* sp*.* |
|  | *Nostocaceae* | Unicell | *Protoperidinium brevipes* | *Evadne* sp*.* | *Synchaeta* sp*.* |
|  | *Crocinitomicaceae* | *Dinobryon* sp*.* | *Heterocapsa* sp*.* | *Acartia bifilosa* | *Centropages hamatus* |

**Table S1.** continued

| Season | Bacteria | Phytoplankton | | Zooplankton | |
| --- | --- | --- | --- | --- | --- |
|  | Abundance | Abundance | Biomass | Abundance | Biomass |
| Summer | *Cyanobiaceae* | *Flagellates* | *Flagellates* | *Evadne* sp*.* | *Podon* sp*.* |
|  | *Ilumatobacteraceae* | *Anabaena cylindrica* | *Mesodinium rubrum* | *Bosmina coregoni* | *Evadne* sp*.* |
|  | *Flavobacteriaceae* | *Chrysochromulina* sp*.* | *Anabaena cylindrica* | *Podon*sp*.* | *Pleopis polyphemoides* |
|  | *Nostocaceae* | *Pyramimonas* sp*.* | *Nodularia spumigena* | *Bosmina* sp*.* | *Bosmina coregoni* |
|  | *Sporichthyaceae* | *Plagioselmis prolonga* | *Aphanizomenon flos-aquae* | *Rotifera* | *Evadne normanni* |
|  | *Rhodobacteraceae* | *Aphanothece paralleliformis* | *Coscinodiscus* sp*.* | *Synchaeta* sp*.* | *Acartia tonsa* |
|  | *Chthoniobacteraceae* | Unicell | *Planktolyngbya* sp*.* | *Pleopis polyphemoides* | *Acartia* sp*.* |
|  | *Pirellulaceae* | *Aphanizomenon flos-aquae* | *Protoperidinium* sp*.* | *Acartia* sp*.* | *Acartia longiremis* |
|  | *Microbacteriaceae* | *Aphanocapsa* sp*.* | *Dinophysis* sp*.* | *Temora longicornis* | *Acartia bifilosa* |
|  | *Burkholderiaceae* | *Chaetoceros* sp*.* | *Peridiniella catenata* | *Acartia longiremis* | *Bosmina* sp*.* |
| Autumn | *Ilumatobacteraceae* | *Flagellates* | *Mesodinium rubrum* | *Acartia* sp*.* | *Evadne normanni* |
|  | *Cyanobiaceae* | *Cylindrotheca closterium* | *Coscinodiscus* sp*.* | *Centropages* sp*.* | *Acartia* sp*.* |
|  | *Sporichthyaceae* | *Plagioselmis prolonga* | *Flagellates* | *Temora longicornis* | *Temora longicornis* |
|  | *Flavobacteriaceae* | *Teleaulax amphioxeia* | *Cylindrotheca closterium* | *Synchaeta* sp*.* | *Centropages hamatus* |
|  | Unknown | *Cyclotella* sp*.* | *Aphanizomenon flos-aquae* | *Appendicularia* | *Appendicularia* |
|  | *Burkholderiaceae* | *Mesodinium rubrum* | *Nodularia spumigena* | *Centropages hamatus* | *Evadne* sp*.* |
|  | *Crocinitomicaceae* | *Chaetoceros* sp*.* | *Centrales* sp*.* | *Fritillaria* sp*.* | *Centropages* sp*.* |
|  | *Thiovulaceae* | *Oocystis lacustris* | *Gyrodinium* sp*.* | *Bosmina* sp*.* | *Pleopis polyphemoides* |
|  | *Rhodobacteraceae* | *Chrysochromulina* sp*.* | *Teleaulax amphioxeia* | *Evadne normanni* | *Fritillaria* sp*.* |
|  | *Pirellulaceae* | *Eutreptiella* sp*.* | *Dinophysis* sp*.* | *Eurytemora* sp*.* | *Acartia tonsa* |

**Table S2.** Seasonal carbon content (mg C m^-3^) in various plankton groups. Values in parenthesis are relative contribution of individual plankton group to the total biomass in percentages. Only samplings when all trophic levels were available are included (n=46).

| Season | Bacterioplankton | Phytoplankton | Litostomatea | Zooplankton | Total biomass |
| --- | --- | --- | --- | --- | --- |
| Winter | 13 (34) | 15 (39) | 8.8 (23) | 1.3 (3) | 38.1 |
| Spring | 18 (15) | 77 (65) | 22 (19) | 1.6 (1) | 118.6 |
| Summer | 44 (30) | 61 (42) | 32 (22) | 9.9 (7) | 146.9 |
| Autumn | 28 (26) | 51 (47) | 27 (25) | 3.2 (3) | 109.2 |
